# Two deletion variants of Middle East respiratory syndrome coronavirus found in a patient with characteristic symptoms

**DOI:** 10.1007/s00705-017-3361-x

**Published:** 2017-04-18

**Authors:** Qian Xie, Yujuan Cao, Juan Su, Jie Wu, Xianbo Wu, Chengsong Wan, Mingliang He, Changwen Ke, Bao Zhang, Wei Zhao

**Affiliations:** 1 Guangzhou Key Laboratory of Drug Research for Emerging Virus Prevention and Treatment, Guangdong Provincial Key Laboratory of Tropical Disease Research, School of Public Health, Southern Medical University, No. 1023 Shatai Road, Guangzhou, 510515 People’s Republic of China; 2 Medical Key Laboratory for Repository and Application of Pathogenic Microbiology, Research Center for Pathogens Detection Technology of Emerging Infectious Diseases, Guangdong Provincial Center for Disease Control and Prevention, Guangzhou, China; 3 Department of Biomedical Sciences, City University of Hong Kong, Hong Kong, China; 4 Guangzhou Key Laboratory of Drug Research for Emerging Virus Prevention and Treatment, School of Public Health, Southern Medical University, Guangzhou, 510515 China

**Keywords:** Infectious Bronchitis Virus, ORF5 Gene, Inverted Repeat Sequence, Deletion Variant, Virus Packaging

## Abstract

Significant sequence variation of Middle East respiratory syndrome coronavirus (MERS CoV) has never been detected since it was first reported in 2012. A MERS patient came from Korea to China in late May 2015. The patient was 44 years old and had symptoms including high fever, dry cough with a little phlegm, and shortness of breath, which are roughly consistent with those associated with MERS, and had had close contact with individuals with confirmed cases of MERS.After one month of therapy with antiviral, anti-infection, and immune-enhancing agents, the patient recovered in the hospital and was discharged. A nasopharyngeal swab sample was collected for direct sequencing, which revealed two deletion variants of MERS CoV. Deletions of 414 and 419 nt occurred between ORF5 and the E protein, resulting in a partial protein fusion or truncation of ORF5 and the E protein. Functional analysis by bioinformatics and comparison to previous studies implied that the two variants might be defective in their ability to package MERS CoV. However, the mechanism of how these deletions occurred and what effects they have need to be further investigated.

**Electronic supplementary material:**

The online version of this article (doi:10.1007/s00705-017-3361-x) contains supplementary material, which is available to authorized users.

## Introduction

Middle East respiratory syndrome coronavirus (MERS CoV) has been reported in more than 23 countries [1] since the first case was identified in 2012 [2]. Infection with this virus leads to a mortality rate of about 40%, but its origin is still not known [3–7]. MERS CoV belongs to lineage C of the betacoronaviruses and has a single-stranded, positive-sense, 30.1-kb RNA genome. The viral genomic RNA encodes four structural proteins, i.e., spike glycoprotein (S), envelope (E), matrix (M) and nucleocapsid (N), as well as several nonstructural proteins, including ORF3-5 and ORF8b [8].

Recently, 186 individuals were confirmed to be infected with MERS CoV in Korea. During the epidemic, one person who was in close contact with a MERS CoV patient started to show MERS symptoms shortly after he traveled to Guangdong Province of China and was confirmatively diagnosed with MERS CoV by lab tests. The patient was cured after 31 days of treatment with antiviral, anti-infection, and immune-enhancing agents. In order to better understand the transmission and evolution of this virus [9], viral RNA was isolated from a nasopharyngeal swab sample of the Korean patient and sequenced. In addition to the wild-type (WT) virus, two deletion variants of MERS CoV were detected in this patient.

## Materials and methods

The cDNA was amplified using 24 pairs of primers (Supplemental Table 1). Each fragment amplified by RT-PCR was about 1500 bp in length. After electrophoresis, PCR products were recovered using a PCR purification kit and sequenced on an AB3730 sequencer (Life Technologies, Guangzhou, China). The sequences obtained from PCR products were assembled into a full-length genome sequence using DNAstar (version 7.0, DNASTAR Inc., Madison, WI, USA). [10]. RNA was extracted from nasopharyngeal swab specimens collected on days 4, 5, 10, and 13 after onset of fever. Reverse transcription of RNA into cDNA was performed as described previously. The cDNA was used as the template for PCR amplification with LA-Taq mix (TaKaRa) and primer pair no. 22. PCR products were analyzed by 1% agarose gel electrophoresis. Protein sequences were aligned using MEGA (version 6.0) [11]. TransMembrane software was used to predict the transmembrane domain of the ORF5 protein (http://www.cbs.dtu.dk/services/TMHMM/) [12]. RNA secondary structure was predicted using RNAfold software, available at http://rna.tbi.univie.ac.at/cgi-bin/RNAfold.cgi [13].

## Results and discussion

**Fig. 1:**
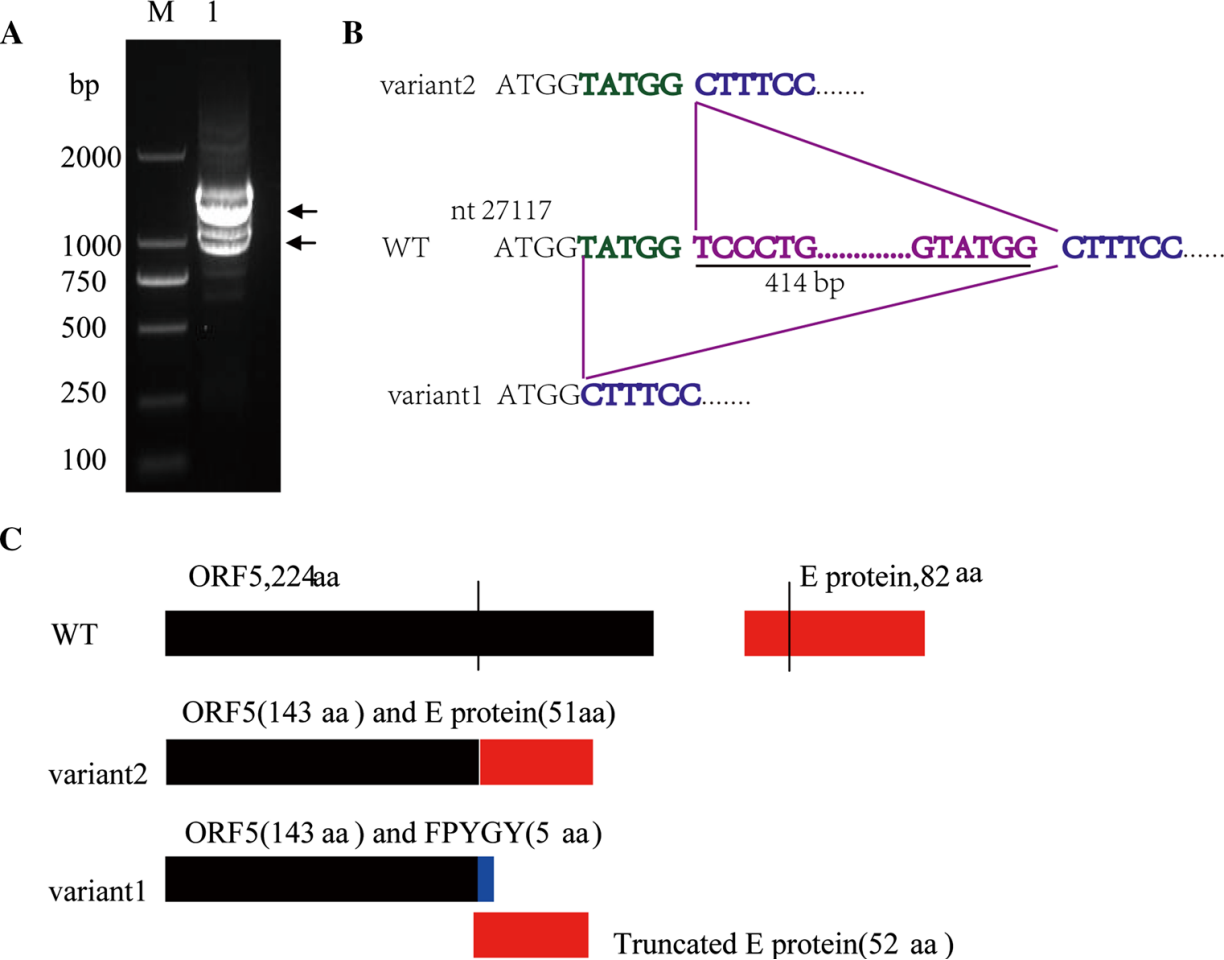
Schematic diagram of WT, variant 1 and variant 2 of MERS CoV. **A.** PCR product electrophoresis of the variant fragment. M, DNA marker; lane 1, no. 22 PCR product of the sample. **B.** Comparison of three sequences in two bands (A). **C.** Protein changes of ORF5 and the E protein in the three genomes (WT, variant 1 and variant 2)

All products yielded usable sequences except those produced using primer pair no. 22. Two specific products obtained by nested PCR (Fig. 1A) were purified, cloned and sequenced. The lower-molecular-weight band was composed of two variants that differed by 5 bp. Variant 2 was longer than variant 1, with the sequence TATGG adjacent to the sequence CTCATGG). The upper band (WT) was 414 bp longer than variant 2 after the sequence CTCATGGTATGG. All fragments of the sequences were assembled into three contigs of WT, variant 1 and variant 2. The genomic sequences have been uploaded to GenBank as KT036372 [14], KT036373 and KT036374, and the main differences in their nucleotide sequences are shown in Fig. 1B.

The predicted changes in the primary structures of the ORF5 and E proteins are shown in (Fig. 1C). Variant 2 encodes a fusion protein of the ORF5 and E proteins (ORF5-E) with an 81-amino-acid (aa) deletion at the C-terminus of ORF5 and a 31-aa deletion at the N-terminus of the E protein. Variant 1 encodes two truncated proteins: a 143-aa fragment of the N-terminus of ORF5 with an additional 5 aa (FPYGY), and a 52-aa fragment of the C-terminus of the E protein. Until now, no such variant has been found in the NCBI database.

**Fig. 2:**
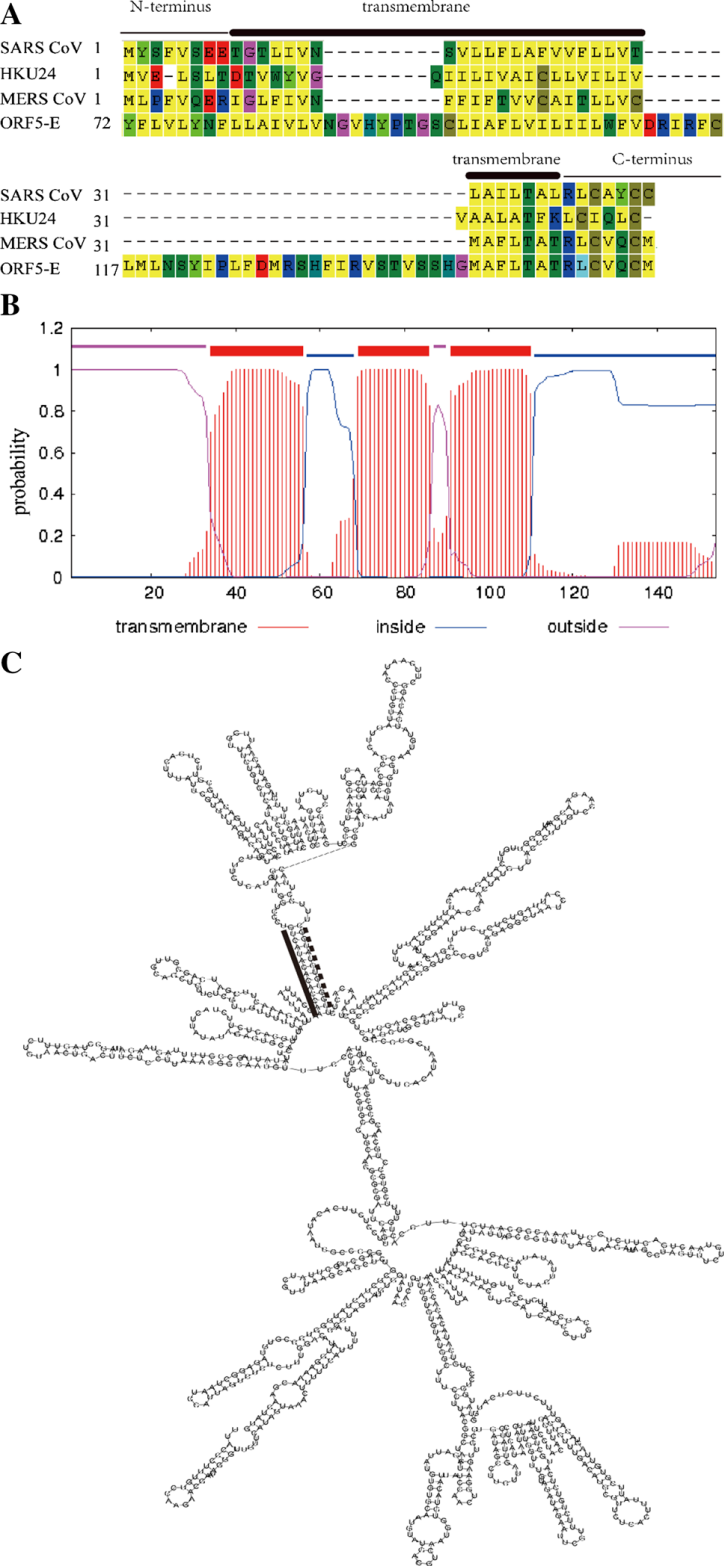
Structure analysis of the E proteins of the wild type and two variants of MERS CoV. **A.** E protein sequence alignment of related coronavirus. The sequences of SARS CoV (NP_828854), HKU24 (NC026011), MERS CoV (NC_019843), and ORF5-E of variant 1 **B.** ORF5 protein transmembrane domain predicted with TMHMM at http://www.cbs.dtu.dk/services/TMHMM/. **C.** RNA secondary structure prediction of the deletion region. RNA secondary structure was predicted using RNAfold software (http://rna.tbi.univie.ac.at/cgi-bin/RNAfold.cgi). The input sequence was based on KT036372, nt 26963-27610. The solid and broken lines indicate the inverted repeat sequence of nt 27131-GTCATACACACCAA-27144 and nt 27527-TTGGTGTGTATGGC-27540, respectively

Although the function of the S protein has been examined previously [15–19], our knowledge of ORF5 and E protein functions in MERS CoV is limited [20]. Moreover, the effects of ORF5 and E protein mutations on viral packaging, infection and disease development have not been evaluated. Based on studies of other coronaviruses, it is believed that the E protein is important for virus packaging and replication [20–22]. The conserved hydrophobic transmembrane N-terminal domain of the E protein is necessary for CoV to be implanted in the membrane. Even single point mutations in the transmembrane protein of the infectious bronchitis virus (IBV) E protein [23], or amino acid changes in the N-terminus of the SARS-CoV E protein can result in attenuation of virulence [24]. To predict the function of the E protein of MERS CoV, we aligned the E and ORF5-E protein sequences of MERS CoV with those of two other coronaviruses, SARS-CoV and China Rattus coronavirus HKU24, using MEGA software (version 6.0) [11]. The results showed that the E protein of MERS CoV shares high similarity with the other two coronavirus (45% for SARS CoV; 60% for HKU24 CoV) in the N-terminal, C-terminal and transmembrane domains (Fig. 2A). The truncated E protein with a deletion of aa 1-30 lacks the N-terminus and a major part of the hydrophobic transmembrane domain in MERS CoV variant 1, which might directly impair virus packaging and replication [24]. The putative fusion ORFF5-E protein (Fig. 2B) encoded by variant 2 is predicted to have three transmembrane regions (TransMembrane Hidden Markov Models [12]), and it remains unclear whether it is able to function like the wild-type E protein.

Almazán et al. reported that MERS CoV with a deletion in the E gene produced replication-competent but propagation-defective virus particles and proposed that this defective virus should be a potential vaccine candidate for preventing MERS CoV infection [25]. The two variants identified in this study carried mutations in the N-terminal domain, which is dispensable for the function of the E protein. However, variations in this region lead to changes in the location of this protein, and therefore, the virulence of these two variants might be impaired to some extent. This needs to be investigated using a recombinant virus.

The ORF5 gene of both variants of MERS CoV in this study was truncated and fused with the E protein. The effect of these variations on the virus could not be predicted because the function of the ORF5 gene is not well understood. However, Scobey et al. found that the effect of ORF5 deletions on the viral replication is minimal, but deletion of the whole ORF5 gene significantly enhances S protein expression [26]. More investigations are required to determine the effects of the ORF5 mutant in these two variants.

Intragenomic sequence deletions have been found in some coronavirus [27, 28]. It has been proposed that this occurs by a copy-choice or template-strand-switching mechanism [29]. One important condition is for there to be a specific leader sequence flanked by the deletion region and a stem-loop structure [30]. Leader sequences corresponding to the UCUAAAC sequence of murine hepatitis virus (MHV) or the CUUAACA sequence of infectious bronchitis virus (IBV) were not found in MERS CoV in this study. Maori et al. have found that inverted repeats facilitate looping out of the middle genomic sequences during RNA replication, resulted in a defective RNA genome [31]. An RNA secondary structure predicted using the RNAfold webserver [13] suggested that the inverted repeat sequence contains long complementary sequences at each end and forms a strong stem-loop structure in the deletion region (Fig. 2C). The deleted sequence was closely adjoined, characterized by a 14-bp nearly complete inverted repeat sequence consisting of 27131-GTCATACACACCAA-27144 and 27527-TTGGTGTGTATGGC-27540, which would result in RNA replicase jumping from one segment to another distant segment. Whether this feature is linked to RNA intramolecular recombination remains to be investigated.

Wild-type MERS CoV and two variants were isolated for the first time from a patient who had traveled from Korea to China. Genomic sequencing revealed 414-bp and 419-bp deletions between ORF5 and the E protein that would result in partial fusion or truncation of these proteins. Whether this finding is a special case or not needs to be investigated by sequencing more samples. Based on previous studies of E protein localization [23–25, 32, 33], we conclude that the two variants might affect virus packaging, which could result in the attenuation of virulence and therefore be relevant for studies related to vaccine development, pathogenesis and viral evolution.

## Electronic supplementary material

Below is the link to the electronic supplementary material.

Supplementary material 1 (DOC 100 kb)
