# Supplementary material for: Two deletion variants of Middle East respiratory syndrome coronavirus found in a patient with characteristic symptoms
Source: Arch Virol. 2017 Apr 18;162(8):2445–9. doi: 10.1007/s00705-017-3361-x (PMC5506503; doi:10.1007/s00705-017-3361-x)
Supplement: Supplementary file 1 — Supplementary material 1 (DOC 100 kb) [file 705_2017_3361_MOESM1_ESM.doc]

Suppl Table1. PCR primers for genomic sequencing of MERS CoV

Primer pair	Forward primer	Reverse Primer	
1	AGCCCTGTTGTTTAGCGTATTGTTG	AACTAACCAACTGAGCAACTGAATG	
2	CCTGGCTTACAGGGAATGCTATC	AGTCGGTACAGCATGACCATTAG	
3	TAGGACTGCTCCAACCTACTGAC	AGTCAAACTCTGTGGAATGTCAAC	
4	GATGCCCGCGCTAAACAGGATG	CACACTGGCATACATATGTCATG	
5	CCAGATGATGCCTCTCGGTTAC	TCAAAGCACTCACTTTAACCTC	
6	TTGCTACCCGCACTTTCACTGC	TTCCAACTTATCGCGTGTGAC	
7	ACTACTACTGGTATACCTGAATAC	CATGTTACCATCATACAAGTCG	
8	TTGCTAATACAGGCAGTGTTTGC	CATTACAACAGTGAATGTACCAG	
9	GATGCCTTGTTGATTTCTATGAC	ATCTGAACTCTTGTGTTGAGACC	
10	GCTTACTCGCCACAGCTTAC	GAGGCAAACTCGGTGTTAGAAC	
11	TCTGATAATGCCTATCTCAAATG	GCAATTTGCGTGATACGTCTG	
12	CAGGAGTAGCTATAGTTGATAG	CATTGACATACAAATCAAACTGC	
13	CAGGTGCTAAGCGAATATGTTC	GCGCCGTCAAGGTAGCCACAG	
14	AGTAAAGTGCAGCTCGGTGA	AAAGCCCATCCTGGAAATAA	
15	TAAGGCAGTCTTTATTTCGC	GCATTATTAGGATATTTTGGTACA	
16	GGCAATCCTAAAGGAATTCCTA	TGACAACCTTGGATACTACGC	
17	CTTCACTTGCTTATTGGTTTATAC	CGACAAACCCATTAGCAAACTG	
18	AAAACTTGGCCTAGGCCAATTG	GCCACCACCTTCAAGTGGAG	
19	AATCCCACATGTTTGATTTTAGCG	ATAAAAGATACTCTGTGCAAATGG	
20	GCAGCAAGGTCCAGCATCAG	GTATAAAGACCAGCTGTATCAGC	
21	TACGACCTCGAGCCGCATAAG	CTTGACGTAGCAGTAATAATAGG	
22	GGCTTTCTCGGCGTCTTTAT	TTTGAGGTGGCCATTGGTTA	
23	AGCTGTTTCAGCTATGATGTGG	CTTGCACCATGTTGAAACTTTTG	
24	CATCTTCAAGAGCCTCTAGCGT	TTGCAAATCATCTAATTAGCCTAATCTAATTG	
